# Quantifying the Food and Physical Activity Environments in Rural, High Obesity Communities

**DOI:** 10.3390/ijerph182413344

**Published:** 2021-12-18

**Authors:** Lacey A. McCormack, Jessica R. Meendering, Linda Burdette, Nikki Prosch, Lindsay Moore, Suzanne Stluka

**Affiliations:** 1 School of Health and Consumer Sciences, South Dakota State University, Brookings, SD 57007, USA; jessica.meendering@sdstate.edu; 2 Formerly College of Nursing, South Dakota State University, Brookings, SD 57007, USA; burdette1323@abe.midco.net; 3 South Dakota State University Extension, Brookings, SD 57007, USA; nikki.prosch@sdstate.edu (N.P.); lindsay.moore@sdstate.edu (L.M.); 4 U.S. Department of Agriculture, National Institute of Food and Agriculture, Washington, DC 20250, USA; suzannestlukard@gmail.com

**Keywords:** obesity, rural, nutrition, physical activity, built environment

## Abstract

The built environment contributes to an individual’s health, and rural geographies face unique challenges for healthy eating and active living. The purpose of this descriptive study was to assess the nutrition and physical activity environments in rural communities with high obesity prevalence. One community within each of six high obesity prevalence counties in a rural Midwest state completed the Nutrition Environment Measures Survey for Stores (NEMS-S) and the Rural Active Living Assessment (RALA). Data were collected by trained community members and study staff. All communities had at least one grocery store and five had at least one convenience store. Grocery stores had higher mean total NEMS-S scores than convenience stores (26.6 vs. 6.0, *p* < 0.001), and higher scores for availability (18.7 vs. 5.3, *p* < 0.001) and quality (5.4 vs. 0, *p* < 0.001) of healthful foods (higher scores are preferable). The mean RALA town-wide assessment score across communities was 56.5 + 15.6 out of a possible 100 points. The mean RALA program and policy assessment score was 40.8 + 20.4 out of a possible 100 points. While grocery stores and schools are important for enhancing food and physical environments in rural areas, many opportunities exist for improvements to impact behaviors and address obesity.

## 1. Introduction

Obesity is a multifaceted public health issue and adult obesity continues to impact much of the United States (U.S.). Data from the National Health and Nutrition Examination Survey (NHANES) indicate that age-adjusted obesity prevalence among U.S. adults aged 20 years and older is 41.9% [1]. Obesity impacts all parts of the country and a variety of demographic groups; however, rural geographies and American Indian populations face distinctive challenges and suffer disproportionately from overweight and obesity. National surveillance data indicate obesity prevalence is significantly higher among rural adults compared to urban adults [2,3]. American Indian or Alaska Native adults are 50% more likely to be obese, and American Indian or Alaska Native adolescents are 30% more likely than non-Hispanic whites to be obese [4].

Environmental features can hinder or support active living and healthy eating environments. The built environment is defined as manmade surroundings that include buildings, public resources, land use patterns, the transportation system, and design features [5]. The built environment’s role in physical activity is a priority for key public health divisions within the Centers for Disease Control and Prevention (CDC) [6] and is a major focus for nutrition, physical activity, and obesity for Healthy People 2020 [7]. There is a growing body of literature that suggests a relationship between physical activity participation and environmental factors, such as the design of the built environment and access to parks, trails, green space, and recreational areas [8,9]. Specific built environment features may influence whether people choose to be active; for example, communities with pedestrian and bicycle-friendly environments can encourage and support walking and biking [10]. Similarly, neighborhoods and individuals living in environments with better access to food retailers, which typically stock fruits, vegetables, and other healthy food options, may have healthier diets and lower levels of obesity than individuals in environments with limited access [11,12,13].

The built environment contributes to an individual’s health, and rural geographies face unique challenges for active living and healthy eating, including isolation, transportation distances, and lack of facilities [14,15]. Unhealthy food consumption, due to limited access to affordable and nutritious food choices, may be more severe in lower-income, rural, minority, and frontier communities [12]. The food environment in rural geographies presents challenges, such as population losses and economic changes that result in limited options for food retail [16]. Adding to these challenges, some of the nutrition and physical activity obesity-prevention strategies recommended for communities by the CDC were found to be less applicable in rural and frontier environments, due to lack of sidewalk networks, transit systems, and government facilities, for example [17]. This highlights a need to assess rural environments to determine their connection with diet and physical activity. In turn, this will help the development and tailoring of interventions that address obesity in these communities. However, there is limited research describing nutrition and physical activity environments in rural and frontier areas with high obesity rates.

In 2014, the Division of Nutrition, Physical Activity and Obesity (DNPAO) at the CDC funded several land-grant universities under the High Obesity Program (HOP) cooperative agreement to reduce obesity prevalence in high-obesity prevalence counties using a community-based, policy, systems, and environmental intervention approach [18]. Recipients were required to conduct needs assessments and plan program activities using these assessments. In South Dakota, this involved quantifying food and physical activity environments in six rural communities to serve not only as a baseline for assessing change over the course of project funding, but also to inform intervention strategies. To address the lack of research in rural areas in general, in addition to the specific needs in South Dakota, the purpose of the present descriptive study was to assess the nutrition and physical activity environments in rural communities with high obesity prevalence to understand whether there are common strengths and challenges that emerge across communities.

## 2. Materials and Methods

Six counties with an obesity prevalence >40% in a rural Midwest state (South Dakota) were identified as part of the CDC HOP funding aimed at improving the nutrition and physical activity environment to support healthy behaviors [19]. One community in each county was recruited to participate in a community-led intervention, which was informed by assessments of the nutrition and physical activity environment. Those assessments are reported here. Communities were chosen based on: access to community and residents (including established connections within the community), proximity to Extension office, having a population substantial enough to form a coalition (population of 400 or more), and limited in Extension work or programming already taking place (highest need). The American Indian population in the six counties ranged from 0.7% to 79%, with an average of 46% [20]. The mean percent of persons living in poverty (based on gross annual income and family unit size) across the six counties was 30.4%, with range of 0.63% to 47.4%. The average community population was 971 residents ranging from 403 to 1963 residents. The mean county area was 1435 square miles with a mean population density of nine persons/square mile. Population density ranged from 1.4 to 31 persons/square mile.

To examine the nutrition environment of each community, the Nutrition Environment Measures Survey for Stores (NEMS-S) [21] was utilized in all grocery and convenience stores. The NEMS-S, a validated observational survey of retail nutrition environments, documents the availability of healthful foods, produce quality, and price differences of healthful vs. less healthful food options in 11 categories, including milk, fruits and vegetables, ground beef, hot dogs, frozen dinners, baked goods, beverages, whole grain bread, baked chips, and cereal. Score ranges are determined for three subtotals: availability (0 to 30 points), price (−9 to 18 points), and quality (0 to 6 points; fruits and vegetables only)) to combine to a total NEMS score range of −9 to 54 points. As an example, stores receive points if ‘lean meats’ are available, and additional points if there are multiple varieties available. Points are added if the price is lower for lean meats vs. others but subtracted if the price is higher for lean meats vs. others. A complete breakdown of scoring and rationale can be found on the tool website (https://nems-upenn.org/tools/, accessed on 29 November 2021). While higher scores are better, there are no cut points for what constitutes an ideal score on NEM-S; rather, the tool can be used to track changes pre/post intervention or examine associations between store environments and eating behaviors [21]. Utilizing nutrition and local content experts, the original tool was modified to include the following additional measures: low-fat milk alternatives, frozen and canned fruits and vegetables, healthier oatmeal, rice, and spaghetti, in addition to canned tuna in water, packaged deli meats (turkey and low-fat bologna), and healthier frozen pizza. Extension personnel identified all grocery and convenience stores to be surveyed, and trained grant personnel subsequently collected data in late 2015.

To examine the physical activity environment of each community, the Rural Active Living Assessment (RALA) [22] was utilized. Data from the RALA include a Town-Wide Assessment (TWA) and a Program Policy Assessment (PPA). The TWA documents demographics and characteristics such as population density, presence and location of a town center, and location of schools. It additionally details the presence of recreational amenities such as walking and hiking trails, biking paths, public parks, swimming pools, recreational centers, and private fitness facilities. The PPA documents programs and policies at the town level such as bikeways and pedestrian walkways in new building projects, policies for clearing snow from sidewalks, the presence of a public recreation department or private organizations that offers physical activity programming, and public transportation options. It further details school programs and polices such as local or national programs that encourage and support active transportation to school, physical activity initiatives, and community access to school recreational facilities. The TWA and the PPA both have a maximum score of 100 points. Maximum section scores vary, ranging from 10 to 30 points across both assessments. There is not an optimal or ideal score. Therefore, data are presented as absolute and relative values allowing for comparison between sections within the TWA and PPA. A group of diverse community stakeholders was trained to utilize the RALA and completed the assessment at a wellness coalition meeting in mid- to late-2015.

Analyses were conducted utilizing IBM© SPSS Statistics Version 24. Descriptive statistics were utilized to compile data on total scores and section scores for the NEMS-S and RALA tools. *T*-tests were used to identify differences in the mean scores for availability, price, and quality of food between grocery stores and convenience stores. Chi-squared tests were used to assess differences in the frequency of availability of healthful food items between grocery and convenience stores. *T*-tests were also used to compare overall RALA TWA and PPA scores. For all analyses, statistical significance was set at *p* ≤ 0.05. Data are presented as mean ± standard deviation.

## 3. Results

### 3.1. Nutrition Environment

All communities had at least one grocery store and five of six had at least one convenience store. Overall NEMS-S scores, in addition to sub-scores for availability, price, and quality for grocery stores and convenience stores by community are presented in Table 1. Grocery stores had significantly higher mean total scores than convenience stores (26.6 vs. 6.0, *p* < 0.001), as well as higher scores for availability (18.7 vs. 5.3, *p* < 0.001), and quality (5.4 vs. 0, *p* = 0.001) of healthful foods. Mean price scores did not vary between grocery stores and convenience stores (*p* = 0.07). Availability of healthful options in both grocery and convenience stores varied by food type (Table 2). Healthful options for milk, soda, juice, bread, and cereal were available in all grocery stores examined, while options for lean ground beef and light or fat-free hot dogs were limited. In general, availability of healthful options among convenience stores was extremely limited; however, diet soda was available in all locations and 100% juice and low fat or skim milk was available in most. While many grocery stores had fresh produce of acceptable quality (85.5% for both fruits and vegetables), fresh fruits or vegetables were unavailable in convenience stores. Overall, healthful options within the categories of milk, fruit, vegetables, frozen dinners, baked goods, bread, and cereal showed greater availability in grocery stores compared to convenience stores (*p* < 0.05).

In terms of pricing of healthful options compared to regular options among the seven grocery stores, healthful options for 100% juice and whole-grain bread were priced lower than regular options in only 14.3% of stores (see Table 2). Healthful options for cereal were priced lower than regular options in 71.4% of stores. In terms of price among convenience stores, only two had a healthful option priced lower than a regular item (milk and/or cereal) and all available whole-grain bread options were priced higher than regular bread.

### 3.2. Physical Activity Environment

The mean RALA town-wide assessment score across communities was 56.5 ± 15.6, ranging from 30 to 74 out of a possible 100 points. Communities scored highest in school location and parks/playground sections, and lowest in the trails section. Recreational facilities and water activities fell in the middle range (Table 3).

The mean RALA program and policy assessment score was 40.8 ± 20.4 ranging from 3 to 56, out of a possible 100 points. Communities strongly supported physical activity through school-based policies and school programming. Town programming scored lower. Town policy held the lowest scoring subscale (Table 3). Total TWA and PPA scores did not differ significantly.

## 4. Discussion

The physical environment in which people live plays a role in shaping both diet and physical activity behaviors, thus ultimately impacting obesity [23,24]. The purpose of this study was to assess the nutrition and physical activity environments in rural communities with high obesity prevalence to understand whether common strengths and challenges emerge across communities. While some strengths were found, many opportunities for improvements in these rural environments exist, especially for addressing obesity.

Nutrition Environment. All communities had a minimum of one grocery store and all but one community had a minimum of one convenience store. Differences were observed, however, between the two types of retail environments in terms of availability (and subsequently quality) of healthy foods. While this was reflected in a higher total NEMS-S score for grocery stores vs. convenience stores (26.6 vs. 6.0, *p* < 0.001), it should be noted that these scores are on a scale of −9 to 54, and grocery stores received only 49% of total possible points. Therefore, although grocery stores are better nutrition environments in these rural areas, they are not necessarily ideal. These findings appear to be in line with others in rural areas. A study examining NEMS-S scores among counties in Montana with varying degrees of rurality based on rural–urban continuum (RUC) codes ranged from 28.7 in the most metro county (RUC code 3) to 21.8 in the most rural county (RUC code 9) with a mean among all counties surveyed of 23.8 (RUC codes 3, 6, 7, 8, 9) [25]. In the present study, RUC codes, which take into consideration population size, urbanization, and adjacency to metro areas [26], ranged from 3 (*n* = 1) to 8 (*n* = 1) to 9 (*n* = 4). For comparison, a study using the NEMS-S in urban Pennsylvania found supermarkets to have total scores approaching 40 points [27]. While physical access to food locations is important, lack of availability of healthful foods within those existing locations compounds the issue [28]. Having access to healthful foods is critical to them being selected [29,30].

As indicated by previous research, the healthfulness of individual’s diets is correlated with the availability of healthful food in stores within the same zip code [31], and rural individuals are less likely to consume the daily recommended servings of fruits and vegetables compared to their urban counterparts [32]. Retail locations, including both grocery and convenience stores, have shown promise as sites for interventions aimed at improving fruit and vegetable consumption, merely by increasing availability [33]. In the present study, however, none of the convenience stores assessed carried fresh fruits or vegetables. While there are grocery stores available within these same communities, it remains unknown how frequently they are visited or whether transportation barriers are present for individuals in accessing grocery stores and their healthier options. Further, although grocery stores carried more healthful options for milk, fruits/vegetables, frozen dinners, baked goods, and cereal, they did not differ in their offerings of healthful beef, hot dogs, bread, and chips relative to convenience stores. Both grocery and convenience stores would benefit from creative solutions to increasing healthful options in these food groups that are culturally appropriate and affordable.

Physical Activity Environment. The RALA town-wide assessment and program and policy assessment scores across communities were 57 and 41 out of 100 possible points, respectively. In the present study, the town-wide assessment ranged from 30 to 74 and the program and policy section ranged from 3 to 56, highlighting wide variation in the amenities, programs, and policies that support physical activity among communities. These data are comparable with two other studies that have previously utilized the RALA to assess the physical activity environment in rural areas. Robinson et al. [34] assessed the physical activity environment in four counties in Alabama and four counties in Mississippi, and found the average town-wide assessment score to be 59 ± 13 with a range of 34–70 and the average programs and policy section score to be 55 ± 24 with a range of 15–86. Perry et al. [35] assessed the physical activity environment using the RALA in four primarily Latino, rural communities in central Washington and found the average town-wide assessment score to be 62.8 ± 13.4 with a range of 47–77 and the average programs and policy section score to be 69 ± 22.5 with a range of 40–95. Common themes that emerged amongst the present study and others [34,35] were that although rural communities have resources that support physical activity, the resources available are often poorly maintained and thus not widely used. Additionally, policies that support physical activity within rural communities require further attention for optimal delivery.

The amenities within the community that emerged as strengths were linked to schools, such as school location within the community and parks and playground availability, as both sections scored ≥78%. The parks and playground section was the least variable (±20%), with all communities scoring a minimum of 15/25 (60%). These data highlight the key role schools play in supporting physical activity within small, rural communities through active transportation to and from school, and the parks and playgrounds associated with the school. The availability of recreational facilities and water activities was less common of an amenity, with the least common amenity section being trails. Only two of the communities had trails, scoring 16/20 with the other four communities earning zero points in this category. These lower scoring sections highlight the opportunity for improvement. While the availability for water activities and trails can be costly to amend, the availability of recreational facilities could be addressed through shared use agreements between the community and the school district to utilize gymnasiums outside of school hours for other recreational needs within the community.

Schools had stronger policies and programs to support physical activity when compared with the community. Although the highest scoring section, the average score in the school policies section was 50%. Only one community earned all points in this category. Since there are national standards for school wellness policies, it was expected to see this section emerge as the highest scoring section but concerning to note the average was only 50%. School programs was the second highest section, highlighting the school as a strength within these rural communities.

Similar to the present findings, school location, parks, and playground amenities were shown to be the highest scoring town-wide assessment sections, and school policy was shown to be the highest scoring program and policy assessment section when assessed in eight communities across Alabama and Mississippi [34], as well as four rural, predominantly Latino communities in central Washington [35]. Together, these data reveal that schools provide a wealth of support for physical activity within rural underserved neighborhoods and efforts should be made to utilize schools to their full potential by maximizing the availability of amenities within schools and further developing programs and policies that support physical activity within schools.

This study is not without limitations. While the NEMS-S is a validated tool, it captures information only about certain locations within communities (i.e., stores), and other places where food is available (i.e., schools or restaurants) were not assessed, nor was the overall availability of food within the community. The RALA is also a validated tool, but it was completed by only one group of stakeholders within the community and could be biased to the information those individuals were aware of and willing to report. Therefore, it is unknown what information remained uncaptured in the assessment. Additionally, while NEMS-S data were collected by Extension professionals, RALA data were collected by community members, which may impact quality of data collection and reporting. Finally, funding limited the type and location of work possible; thus, there remains a small number of communities to assess, and the study is descriptive in nature. The findings presented are specific to rural communities and are unlikely to be generalized to other geographic locations.

## 5. Conclusions

Conducting assessments on the nutrition and physical activity environments within rural communities is helpful in identifying existing supports that can be enhanced, and in identifying barriers and challenges to overcome. This facilitates interventions that are feasible and impactful for communities while optimizing the use of existing resources. In the rural communities examined in the present study, nutrition environments are enhanced by grocery stores—although price and overall availability of healthful food requires further improvement—and schools serve as an important physical activity hub. This information will be used for developing and implementing community-based, obesity prevention interventions, tailored for rural communities.

## Figures and Tables

**Table 1:** Overall NEMS-S scores and sub-scores for grocery and convenience stores by community.

	Total (−9 to 54)	Availability (0 to 30)	Price (−9 to 18)	Quality (0 to 6)
*Grocery Stores*				
Community 1	25	19	0	6
Community 2	28	18	4	6
Community 3	28	19	3	6
Community 3	8	8	0	0
Community 4	30	24	0	6
Community 5	35	22	7	6
Community 6	32	21	5	6
Mean(SD)	26.6 (8.8)	18.7 (5.2)	2.7 (2.8)	5.4 (2.3)
*Convenience Stores*				
Community 1	4	4	0	0
Community 2	5	6	−1	0
Community 3	12	10	2	0
Community 3	8	6	2	0
Community 4 ^1^	^-^	^-^	^-^	^-^
Community 5	5	3	2	0
Community 5	7	8	−1	0
Community 5	6	4	2	0
Community 5	2	2	0	0
Community 6	5	5	0	0
Mean(SD)	6 (2.8)	5.3 (2.5)	0.7 (1.3)	0 (0)
				
*p*-value ^2^	<0.001	<0.001	0.07	<0.001

^1^ *n* = 0. ^2^ *t*-tests comparing total mean score between grocery and convenience stores.

**Table 2:** Comparison of availability of healthful food options between grocery and convenience stores and pricing for healthful vs. regular food options in grocery stores and convenience stores (NEMS-S).

	Grocery Store (%) *n* = 7	Convenience Store (%) *n* = 9	*p*-Value
*Availability of Healthful Options*			
Low-fat/skim milk ^1^	100	77.8	0.012
# of fresh fruit varieties ^2^			0.003
0	14.2	100	
1–5	0.0	0.0	
5–9	28.6	0.0	
10	57.1	0.0	
# of fresh vegetable varieties ^2^			0.003
0	14.2	100	
1–5	0.0	0.0	
5–9	0.0	0.0	
10	85.7	0.0	
Lean ground beef (≤10% fat) ^2^			0.341
1 variety	28.6	0.0	
2 varieties	14.2	0.0	
>3 varieties	0.0	0.0	
Hot Dogs			0.341
Fat-free	0.0	0.0	
Light, not fat-free	28.6	0.0	
Frozen Dinners ^2^			0.013
All 3 Reduced-fat types	14.2	0.0	
1 or 2 Reduced-fat types	71.4	11.1	
Low-fat baked goods ^1^	71.4	0.0	0.012
Diet soda ^1^	100	100	-
100% juice ^1^	100	88.9	-
Whole grain bread ^2^			0.27
1 variety	100	22.2	
>2 varieties whole wheat bread	28.6	0.0	
Baked chips ^2^			0.154
1 variety	57.1	0.0	
>2 varieties	0.0	0.0	
Healthier cereal ^1^	100	22.2	0.009
*Healthful Options Priced Lower than Regular Options*			
Lowest fat milk	28.6	22.2	
Lean ground beef	0	0	
Reduced-fat or light hot dogs	14.3	0	
Reduced fat frozen dinners	14.3	0	
Low-fat baked goods	42.9	0	
Diet Soda	14.3	0	
100% Juice	14.3	0	
Whole Grain Bread	14.3	0	
Baked Chips	14.3	0	
Cereal	71.4	11.0	

^1^ *t*-tests comparing total mean score between grocery and convenience stores. ^2^ Chi-squared tests comparing frequencies of responses between grocery stores and convenience stores.

**Table 3:** Town-wide and program and policy assessment section scores by community (RALA).

Community	1	2	3	4	5	6	Absolute Section Scores Mean ± SD	Relative Section Scores ^1^ Mean ± SD
*Town-wide Assessment*								
School Location (0–15)	0	15	15	15	15	15	12.5 ± 6.1	83.3 ± 40.8
Trail (0–20)	0	16	0	0	16	0	5.3 ± 8.3	26.7 ± 41.3
Parks and Playgrounds (0–25)	16	23	25	15	25	15	19.8 ± 5.0	79.3 ± 20.0
Water Activities (0–10)	5	0	10	5	5	0	4.2 ± 3.8	41.7 ± 37.6
Recreation Facilities (0–30)	28	9	17	21	13	0	14.7 ± 9.7	48.9 ± 32.4
Total Score (0–100)	49	63	67	56	74	30	56.5 ± 15.6	56.5 ± 15.6
*Program and Policy Assessment*								
Town Policies (0–10)	0	3	0	3	10	0	2.7 ± 3.9	26.7 ± 38.8
Town Programs (0–30)	16	0	8	18	0	12	9.0 ± 7.8	30.0 ± 25.9
School Policies (0–30)	30	0	15	15	15	15	15.0 ± 9.5	50.0 ± 31.6
School Programs (0–30)	10	0	10	10	30	25	14.2 ± 11.1	47.2 ± 37.1
Total Score (0–100)	56	3	33	46	55	52	40.8 ± 20.4	40.8 ± 20.4

^1^ Calculated as a percent: (absolute score/points possible) ∗ 100.

## Data Availability

The data presented in this study are available on request from the corresponding author. The data are not publicly available due to restrictions limiting sharing of data to state-level data by request.
